# New Insights into the Immune Molecular Regulation of the Pathogenesis of Acute Respiratory Distress Syndrome

**DOI:** 10.3390/ijms19020588

**Published:** 2018-02-16

**Authors:** Chin-Yao Yang, Chien-Sheng Chen, Giou-Teng Yiang, Yeung-Leung Cheng, Su-Boon Yong, Meng-Yu Wu, Chia-Jung Li

**Affiliations:** 1 Division of Chest Medicine, Kaohsiung Veterans General Hospital, Kaohsiung 813, Taiwan; ycyaoyao@vghks.gov.tw; 2 Department of Emergency Medicine, Taipei Tzu Chi Hospital, Buddhist Tzu Chi Medical Foundation, New Taipei 231, Taiwan; holeyeye@yahoo.com.tw (C.-S.C.); gtyiang@gmail.com (G.-T.Y.); 3 Department of Emergency Medicine, School of Medicine, Tzu Chi University, Hualien 970, Taiwan; 4 Division of Thoracic Surgery, Department of Surgery, Taipei Tzu Chi Hospital, Buddhist Tzu Chi Medical Foundation, New Taipei City 231, Taiwan; ndmc0928@yahoo.com.tw; 5 School of Surgery, Tzu Chi University, Hualien 970, Taiwan; 6 Institute of Medicine, Chung Shan Medical University, Taichung 402, Taiwan; yongsuboon@gmail.com; 7 Division of Pediatric Allergy, Immunology and Rheumatology, Department of Pediatrics, Show Chwan Memorial Hospital, Changhua 500, Taiwan; 8 Department of Nursing, Meiho University, Pingtung 912, Taiwan; 9 Research Assistant Center, Show Chwan Memorial Hospital, Changhua 500, Taiwan

**Keywords:** acute respiratory distress syndrome, vascular permeability, NETosis, sepsis

## Abstract

Acute respiratory distress syndrome is an inflammatory disease characterized by dysfunction of pulmonary epithelial and capillary endothelial cells, infiltration of alveolar macrophages and neutrophils, cell apoptosis, necroptosis, NETosis, and fibrosis. Inflammatory responses have key effects on every phase of acute respiratory distress syndrome. The severe inflammatory cascades impaired the regulation of vascular endothelial barrier and vascular permeability. Therefore, understanding the relationship between the molecular regulation of immune cells and the pulmonary microenvironment is critical for disease management. This article reviews the current clinical and basic research on the pathogenesis of acute respiratory distress syndrome, including information on the microenvironment, vascular endothelial barrier and immune mechanisms, to offer a strong foundation for developing therapeutic interventions.

## 1. Introduction

Acute respiratory distress syndrome (ARDS) is a life-threatening lung condition that leads to hypoxia by interfering with the delivery of oxygen from alveoli into the blood [1]. In 1967, the term “adult respiratory distress syndrome” was first promoted by Ashbaugh et al. to describe the condition in 12 patients [2]. Subsequent recognition that this lung condition occurred in patients of all ages led to the coining of the current term, in which “acute” replaced “adult.” Several conditions can induce ARDS, such as severe pancreatitis, massive blood transfusion, severe sepsis, pneumonia, and mechanical ventilation [3,4,5,6,7], by damaging epithelial and/or endothelial cells and inducing inflammation. Endothelial dysfunction and local inflammation cause diffuse alveolar injury, leading to bilateral pulmonary infiltrates and severe hypoxemia [8,9,10,11]. Severe lung injury may develop into respiratory distress and respiratory failure over the course of hours to days. ARDS is associated with high mortality and morbidity rates, which increase with disease severity [12,13,14,15,16]. Despite years of basic and clinical studies, the detailed pathophysiology of the microvascular dysfunction and the micro-inflammatory responses in ARDS remain unclear, especially with respect to the molecular regulation of the immune response. In this review article, we analyze the current basic and clinical studies to offer an overview of the vascular permeability molecular regulation and microenvironment in ARDS. We also summarize the mechanisms of ARDS to provide a strong foundation for the development of novel treatment approaches.

## 2. Epidemiologic and Clinical Features

**Figure 1:**
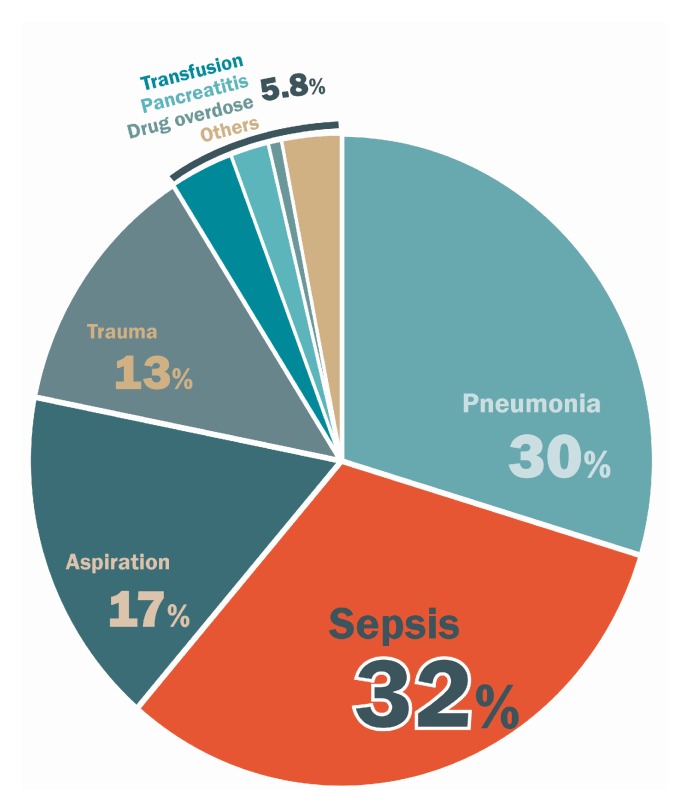
The etiology of acute respiratory distress syndrome (ARDS) (adapted from Bersten et al. [13]).

The incidence of ARDS varies by geographical location and population. In a multicenter prospective cohort study, the age-adjusted incidence estimates ranged from 64 to 86 per 100,000 person-years for moderate to severe ARDS [16]. The rate of ARDS-related mortality increases with the severity of lung injury. A multicenter prospective cohort study by Bellani et al. reported that the rate of hospital mortality was 34.9% in patients with mild ARDS, 40.3% for those with moderate ARDS, and 46.1% for those with severe ARDS [12]. The underlying cause of ARDS is a critical determining factor of the mortality rate. Patients with ARDS rarely die due to respiratory failure alone. In the Bersten et al. [13] study, pneumonia and sepsis were the most common causes of death, accounting for 30% and 32% of deaths, respectively. Other etiologies of ARDS accounted for 38% of deaths, including aspiration (17%), trauma (13%), transfusion (3.3%), pancreatitis (2%), and drug overdose (0.7%) (Figure 1). Infection was a major cause of death in ARDS patients. Severe sepsis is a critical condition caused by inflammatory cascades in response to infectious pathogens [17]. The general inflammatory status also affects, not only ARDS, but hypotension and hypoperfusion of multiple organs. It is especially important to understand the relationship between sepsis and ARDS. Timely treatment of sepsis and prevention of the vicious cycle of ARDS can help to decrease morbidity and mortality.

The clinical features of ARDS progress rapidly within 72 h, resulting in respiratory distress and bilateral alveolar infiltrates [18] that cannot be attributed to cardiogenic causes [19]. In 1994, the American-European Consensus Conference (AECC) proposed the first set of clinical diagnostic criteria for ARDS based on its clinical features (Table 1) [20]. However, the AECC criteria were not clear, due to the inadequate definition of the timing of the disease, poor reliability of image interpretation, and inconsistency in the ratios of the arterial oxygen tension (PaO_2_) to the inspiratory oxygen fraction (FiO_2_). In 2012, the Berlin definition [21] was promoted, with clearer definitions that refined the AECC criteria. The Berlin criteria had improved predictive ability for ARDS-related mortality over the AECC definition [22].

## 3. Overview of Pathogenesis of ARDS

**Figure 2:**
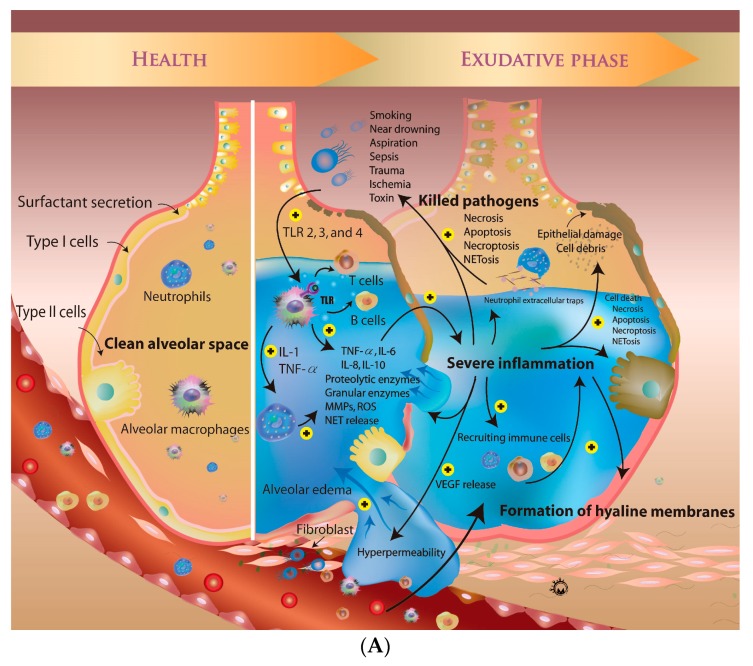

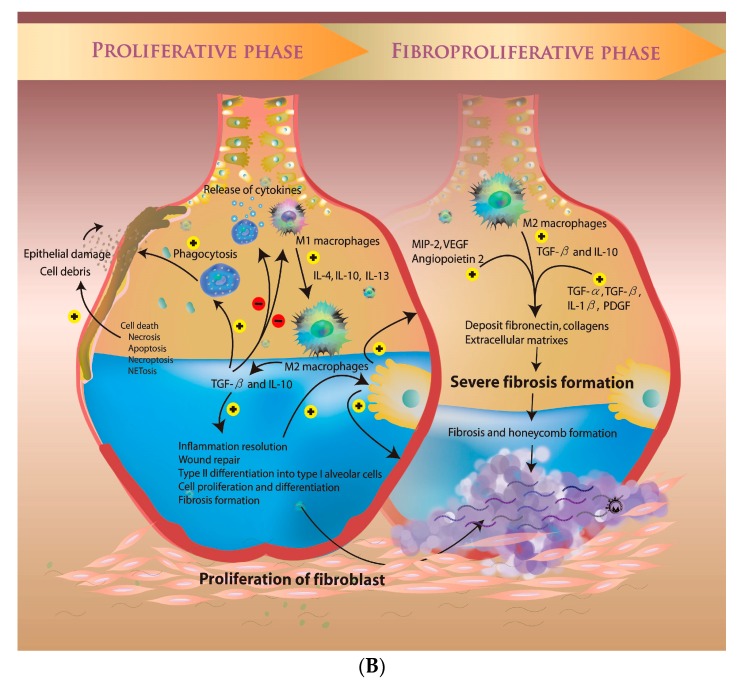
The molecular regulation of the pathogenesis of acute respiratory distress syndrome in four major phases: (**A**) Health and exudative phase (**B**) proliferative phase and fibroproliferative phase.

ARDS is the result of an initial acute systemic inflammatory response due to direct or indirect lung injury, caused by factors such as smoking, near-drowning, aspiration, sepsis, trauma, ischemia, and exposure to a toxin [23,24]. The severe inflammatory responses induce the change of vascular permeability, leading to acute pulmonary edema. There are three major phases of ARDS: the exudative, proliferative, and fibroproliferative phases [25]. In the exudative phase, the lung injury-induced inflammation cascade increases epithelial permeability, causing diffuse alveolar edema. Hyaline membranes form in the proliferative phase, accompanied by the infiltration of inflammatory cells, including T cells, neutrophils, and macrophages. After severe damage caused by inflammation and oxidative stress, the extracellular matrix deposited at alveoli accompanied with persisted chronic inflammation. Inflammatory cascades play key roles in processes that are closely involved in ARDS, such as cell apoptosis, proliferation, and migration. Dense fibrosis and a honeycomb-like structure can be detected in fibroproliferative phases of ARDS with imaging studies, such as computed tomography scans or chest X-rays. Due to vascular endothelial and alveolar epithelial damage, the capacity for gas exchange decreases, leading to acute respiratory failure, which may require ventilatory and critical care support. The catastrophic illness associated with ARDS carries a high risk of ventilator-acquired pneumonia, acute myocardial infarction, and acute pulmonary embolism. Below, we provide an overview of the recent advances in the knowledge of the cellular and molecular biology of ARDS, with an aim to facilitate the development of future disease-modifying therapies (Figure 2).

### 3.1. Mechanisms of Regulation of Vascular Permeability

The healthy alveolar epithelium is comprised of two types of cells that form a natural barrier. Type I cells, which make up 90% of the alveolar epithelium, are sensitive to toxins and oxidative stress. They are easily injured compared to type II cells, which make up 10% of the alveolar surface area. Type II cells participate in surfactant production and sodium transport, and induce proliferation and differentiation to type I cells. Type II cells also play an important role in innate immunity due to their expression of Toll-like receptors 2 and 4 [26]. The production of surfactant prevents atelectasis and sodium transport keeps fluid out of the alveolus. These functions protect the alveolar microenvironment. Another barrier between the alveolus and capillaries is the microvascular endothelium. Endothelial cells regulate vasoconstriction and dilation via nitrogen oxide formation [27] and also mediate vascular permeability to prevent pulmonary edema by selective transport of fluids, electrolytes, and macromolecules via dynamic opening of the intercellular junctions [28]. The interaction between the 2 separate barriers plays a critical role in controlling fluid accumulation in the alveolar space (Figure 2). Various molecular pathways also regulate vascular permeability, such as those involving sphingosine-1-phosphate (S1P), thrombin, and angiopoietin 1 and 2 [29]. S1P, a blood lipid mediator, binds to its receptor, S1P1, to regulate vascular permeability via a Rac-dependent pathway [29,30]. The receptor of thrombin can be cleaved to increase Rho/ROCK signaling, leading to hyperpermeability. Angiopoietin 1 and 2 bind to tyrosine kinase with immunoglobulin-like and EGF-like domains 2 to regulate vascular permeability via the activation of Rho A [31,32].

Lung injury can occur by several mechanisms, especially sepsis. The immune response is triggered by activation of antigen-presenting cells (APCs), such as monocytes, macrophages, dendritic cells, and endothelial cells. These cells respond to pathogens or toxins. After interaction with pathogens or specific pathogen-derived components, APCs present the pathogen-associated molecular patterns to T cells. This signaling activates immune cells to secrete inflammatory mediators, such as tumor necrosis factor (TNF) α, interleukin (IL)-1, IL-6, IL-8, prostaglandins, and histamine [33,34]. The inflammatory signaling can be amplified, leading to vascular endothelium dysfunction and the influx of more neutrophils, monocytes, macrophages, and lymphocytes. This causes vascular permeability to increase, accompanied by vasodilatation, via overproduction of nitric oxide by endothelial cells. Severe systemic inflammation-induced lung injury via pulmonary microvascular hyperpermeability is the first step in the development of ARDS. Exudation from the plasma to the alveolar spaces decreases alveolar fluid clearance, leading to lung edema. Severe inflammation also activates infiltrating leukocytes to release potent cytotoxic mediators, such as proteolytic enzymes, granular enzymes, reactive oxygen species (ROS), cytokines, chemokines, and neutrophil extracellular traps (NETs), which minimize the damage to the host when neutrophils kill pathogens. The severe inflammation causes losses of endothelial and epithelial cells by inducing apoptosis and necrosis. Alveolar epithelial cells downregulate ion transport machinery and the production of vascular endothelial growth factor (VEGF), leading to alveolar edema. In addition, gas exchange is blocked by the accumulation of free fluid.

### 3.2. Microenvironment during the Proliferative Phase

In the proliferative phase, immune cells are recruited to and accumulate in the alveolar space through the capillaries. The release of VEGF, a permeability factor, also plays an important role in the pathogenesis of ARDS by promoting vascular permeability, exudation of protein-rich fluid, and migration of inflammatory cells [35]. A rise in the plasma levels of VEGF causes free fluid to move through the capillary wall into the alveolar space [35,36,37]. A large number of inflammatory mediators are also released, which cause endothelial cell damage. Apoptosis signaling is activated via the extrinsic and intrinsic pathways. The death ligands, such as TNF-α, TNFSF12 (TWEAK), Fas ligand, TNFSF10 (TRAIL), and TNFSF15 (TL1A), activate the extrinsic pathway to induce cell death via the caspase cascade [38]. The ROS from cytokines also activate DNA and mitochondrial damage [39]. The change in the integrity of the mitochondrial membrane activates the pro-apoptotic Bcl-2 family to regulate downstream pro-apoptotic proteins, such as cytochrome c, and cause apoptosis via the intrinsic pathway [40]. In addition, increasing ROS levels induce necroptosis in the setting of ARDS [41,42,43]. Necroptosis signaling activates the formation of the necrosome via receptor-interacting protein kinase 3 (RIP3) and mixed lineage kinase domain-like protein (MLKL). PARP activation due to DNA damage also leads to necrosome formation. Dead cell debris and protein-rich fluid accumulate in the alveolar space to form the hyaline membrane, which is a characteristic pathological finding in the proliferative phase of ARDS. The presence of hyaline membranes decreases the capacity for gas exchange due to acute lung edema. To recover from lung injury, type II alveolar cells differentiate into type I alveolar cells (Figure 2). The formation of new vascular endothelial cells improves oxygenation via the recovery of microvascular permeability.

### 3.3. The Fibroproliferative Phase of ARDS

In up to 30–50% of adult patients, the inflammatory response fails to be resolved in the fibroproliferative phase, leading to the development of fibrosing alveolitis with cystic changes and the limitation of pulmonary function by infiltrating macrophages, fibrocytes, fibroblasts, and myofibroblasts. Many pro-fibrotic agents are released, such as transforming growth factor (TGF) α, TGF-β, IL-1β, and platelet-derived growth factor, which causes an imbalanced response to anti-fibrotic mediators, including prostaglandin E_2_ and other growth factors, leading to the deposit of excess fibronectin, collagens, and other extracellular matrix components [44]. The overexpression of angiogenic cytokines and growth factors by immune cells, including macrophage inflammatory protein 2, angiopoietin 2, and VEGF, may drive the fibroproliferative response (Figure 2) [45,46,47,48]. Inadequate mechanical ventilation used to improve gas exchange may also induce fibroproliferation. The shear forces from inadequate mechanical ventilation injure the lung and promote acute and chronic fibroproliferation [49,50,51,52]. Persistent, severe damage to the lung structure impairs cellular repair and promotes pathological fibroproliferation. The latest phases of ARDS are characterized by a remodeled lung architecture marked by fibrosis and honeycomb formation [2,25].

In healthy alveoli, the two types of epithelial cell and alveolar macrophage form a natural barrier. When direct or indirect lung injury occurred, the macrophage and neutrophil are activated by Toll-like receptors. Macrophages produce TNF-α, IL-1, IL-6, IL-8, IL-10 and recruit neutrophils. The neutrophils release ROS, several enzymes and cytokines. In exudative phase, the severe inflammation lead to higher concentration of VEGF, leading to alveolar edema. The local inflammation also recruits other immune cells and kills pathogens via NETosis, necrosis, apoptosis and necroptosis. Severe inflammatory responses also cause both types alveolar epithelial cells death. In proliferative phase, dead cell debris and protein-rich fluid accumulate in the alveolar space to form the hyaline membrane. The inflammatory reactions resolve via the transition from M1 to M2 macrophages. The M2 macrophages promote wound repair, alveolar epithelial cell transition, and fibrosis formation by release of TGF-β and IL-10. In addition, several growth factors, such as MIP-2, angiopoietin, VEGF, and PDGF, also promote the deposition of fibronectin and collagens. The severe fibrosis in fibroproliferative phase leads to lung architecture remodeling marked by fibrosis and honeycomb formation.

## 4. The Micro-Inflammatory Response of ARDS

ARDS is an acute inflammatory disease caused by infectious insult. Pathogens, such as viruses, bacteria, and other microorganisms, are believed to induce pulmonary cell injury, leading to ARDS. Although the detailed mechanisms of ARDS resulting from sepsis or pneumonia remain unknown, the innate immune system is known to play a profound role in the pathophysiology of ARDS [53]. Severe inflammatory responses are induced by pathogens via mediators such as toxins and pathogen-associated molecular patterns. The resulting cell apoptosis, necrosis, and necroptosis produce damage-associated molecular patterns, which lead to the release of heat shock proteins and, ultimately, activate immune reactions [1]. When intracellular innate immune mechanisms fail to control the early infectious process, APCs are activated and phagocytosed the infected cells. Alveolar macrophages are important in the activation of inflammatory cascades. They recruit other immune cells to the injury site and release many pro-inflammatory mediators [1].

### 4.1. Role of Alveolar Macrophages in ARDS

When alveolar macrophages are exposed to infectious agents, they activate the immune response upon recognition of Toll-like receptor ligands, pathogen-associated molecular patterns, and danger-associated molecular patterns [53]. The activated alveolar macrophages can be divided into two main phenotypes: M1 and M2 macrophages [54]. The M1 macrophages encourage inflammation by secreting pro-inflammatory cytokines, such as IL-1β, IL-12, TNF-α, IFN-γ, IFN-β, and inducible nitric oxide synthase. M2 macrophages contribute to tissue repair due to their anti-inflammatory functions, mediated by releasing Th2 cytokines, such as IL-4, IL-10, and IL-13 [55,56]. Circulating monocytes and macrophages are recruited to the alveolar space and are activated by macrophage colony-stimulating factor mediators produced by T cells, macrophages, endothelial cells, and fibroblasts. After they phagocytose apoptotic cells, M1 macrophages promote T cell proliferation by interaction of macrophage-derived CD80/86 and MHC-II with CD28 and T cell receptors, respectively. The interaction also induces neutrophil, macrophage, and monocyte recruitment. M1 macrophages release toxic species, such as nitric oxide, superoxide, and matrix metalloproteinases that cause tissue damage. The TNF-α and IL-1β produced by macrophages also activate neutrophils and induce the overexpression of adhesion molecules, such as intercellular adhesion molecule 1 and vascular cell adhesion molecule 1, on immune cells and endothelial cells [57,58,59]. The effects of TNF-α and IL-1β in sepsis are amplified, leading to the recruitment of more inflammatory cells into the alveolar injury site. High concentrations of TNF-α and IL-1β have been reported in the bronchoalveolar lavage fluid of ARDS patients [60,61].

In addition, M1 macrophages transited to M2 macrophages due to the expression of IL-4, IL-10, and IL-13. After pathogen clearance, anti-inflammatory responses mediated by M2 macrophages resolve local inflammation and pulmonary injury. M2 macrophages abrogate pro-inflammatory mediators, induced to augment of TGF-β and IL-10, and promote resolution of the immune response and wound repair. TGF-β regulates cell proliferation, differentiation, apoptosis, and necroptosis. It also plays an important role in the resolution of pulmonary inflammation and fibrosis formation [62,63,64,65]. IL-10 is an anti-inflammatory cytokine that prevents subsequent pulmonary damage by inhibiting the production of pro-inflammatory cytokines, such as IL-1β, IL-6, and TNF-α, by macrophages [66,67,68,69]. In clinical studies, low levels of IL-10 but high levels of TNF-α were reported in the bronchoalveolar lavage fluid of ARDS patients [70,71]. The concentrations of IL-10 and TNF-α in the lung reflect the balance of pro-inflammatory versus anti-inflammatory activity in ARDS.

### 4.2. Activated Neutrophils in ARDS

In ARDS, neutrophils are considered an important component of the inflammatory microenvironment. Several chemokines in the pulmonary injury site, such as IL-8 and IL-17, regulate neutrophil recruitment from vessels into the alveolar space, leading to tissue damage and alveolar–capillary hyperpermeability by releasing toxic ROS and cytokines. In a clinical study, a higher concentration of neutrophils was associated with more severe inflammation, hypoxia, higher permeability, and poor outcome [72]. Activated macrophages release IL-8 and CXCL5, thereby promoting the activation of neutrophils. The lymphocytes also release potent neutrophil chemoattractants, such as IL-1, in sepsis-induced ARDS, resulting in the accumulation of neutrophils in the alveolar space. In in vitro studies, several factors associated with neutrophil recruitment have been reported, including keratinocyte-derived chemokine, cytokine-induced neutrophil chemoattractant, macrophage inflammatory protein 2, CXCL1, CXCL2, and lipopolysaccharide-induced CXC chemokine [73,74,75,76,77]. Excessive neutrophil recruitment occurs in ARDS via alteration of the CD62L expression levels upon decreases in the de-priming mechanisms [78]. After activation, neutrophils that infiltrate alveolar spaces engulf microbes and secrete anti-microbial cytokines and chemokines. Cytokines can activate the endothelium and recruit other circulating leukocytes [79].

The release of 4 types of secretory vesicles from neutrophils—azurophilic (primary), specific (secondary), gelatinase (tertiary), and secretory vesicles—promotes severe local inflammatory at the onset of ARDS. High levels of serine proteases from neutrophils have been reported in the bronchoalveolar lavage fluid and plasma of ARDS patients. The serine proteases and elastases promote the production of pro-inflammatory cytokines by epithelial cells and induce cell apoptosis via the PAR-1-, NF-κB-, and p53-dependent pathways [80,81,82,83]. In recent reports, the release of secretory vesicles and ROS from neutrophils induced the formation of NETs to cause NETosis [84]. The binding of alveolar pathogens to neutrophil surface receptors increases the level of cytoplasmic calcium via release from the ER and the opening of membrane channels. The accumulation of calcium activates PKC activity and induces ROS formation from NADPH and nitric oxide production. The oxidative stress leads to breakage of the nuclear and granule membranes and the release of NETs. NETs, extracellular fibers networks composed of neutrophil DNA with antimicrobial proteins, were first described in 2004 [85]. NETs bind to extracellular pathogens with minimal damage to host cells. They form protective structures to prevent infection progression in sepsis-induced ARDS. Neutrophil-derived ROS from NADPH oxidase and nitric oxide synthase also induce pulmonary injury in ARDS. Several basic and clinical studies reported that higher tissue levels of ROS led to histological changes in the setting of lung injury and increased alveolar–capillary permeability. ROS also cause endothelial and epithelial cell apoptosis and impair gas exchange.

### 4.3. Role of T and B Cells in ARDS

After APC activation, the cells recruit circulating T and B cells to cause inflammatory cascades. Endothelial cells release IL-16 to recruit CD4^+^ cells and prevent neutrophil recruitment via IL-10. T and B cells are also activated by bacterial lipopolysaccharides, bacterial fragments, or viral DNAs and RNAs, by binding to their surface receptors. They release anti-pathogenic, inflammatory agents, such as interferons and interferon-related proteins, and pro-inflammatory cytokines [1]. However, there are few studies published on the roles of B and T cells in the pathophysiology of ARDS. In an indirect acute lung injury study, acute lung injury was induced in mice lacking B and T cells. The pulmonary injury and cell apoptosis in the deficient group were higher than in the control group. The B and T cells, therefore, play protective roles. A deficiency in cytotoxic CD8^+^ T cells was also associated with increased pulmonary cell apoptosis. These results are interesting, but require additional clinical and basic investigation and confirmation.

## 5. Vascular Endothelial Barrier and Vascular Permeability

Endothelial cells as the basic structure and function unit of vascular endothelium, one of its important functions is to play its barrier function, together with the extracellular matrix constitutes a complete endothelial cell barrier, regulate the exchange of substances inside and outside the blood vessels, to maintain the stability of the internal environment, making tissues and organs near or far from damage. The integrity of the vascular endothelial barrier relies on both cellular attachment and myosin contractility. Among them, cellular connections mainly include adhesive connections and tight connections. Adhesive junctions form a complex of VE-cadherin and catenin (β-catenin, γ-catenin and p120-catenin); tight junctions consist of proteins such as the transmembrane proteins claudins and ZO-1. Under pathological conditions, the destruction of cell junctions promotes the formation of intercellular cracks. Thus, the combined effect of cell–cell junctions and impaired cell-cell intercellular connections is an important cause of increased vascular permeability [86].

### 5.1. Role of Rho/ROCK Signaling Pathway

**Figure 3:**
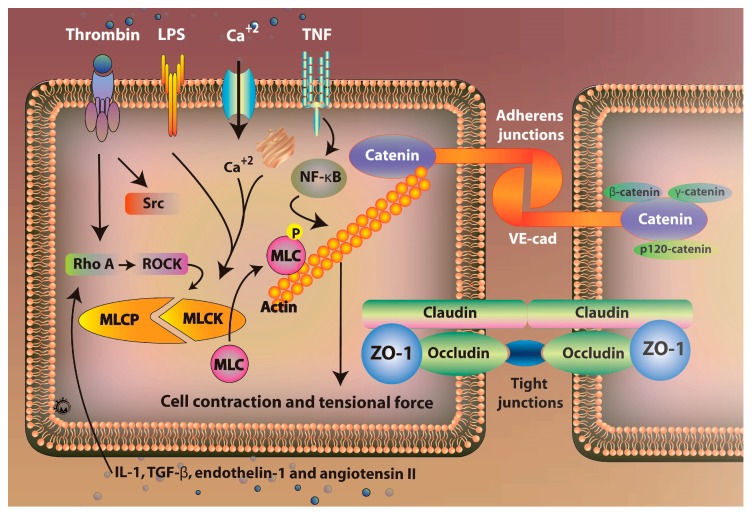
Mechanisms of pulmonary vascular endothelial regulation. Vascular permeability was regulated via actin-myosin interaction. The barrier disruption and enhancement was mediated by the phosphorylation of myosin light chain (MLC) regulated by myosin light chain kinase (MLCK) and myosin light chain phosphatase (MLCP). Activation of the actin myosin increases stress fiber formation and cause in cell contraction and tensional force to adherens junction (AJ). Rho/ROCK signaling can be activated by thrombin, IL-1, TGF-β, endothelin-1 and angiotensin II. Activation of RhoA induced ROCK to activate MLCK, causing to enhance vascular permeability. The MLCK also is activated by Ca^2+^ from endoplasmic reticulum or extracellular space. The TNF and Ca^2+^ may induced expression of nuclear factor κB (NF-κB) causing to degrade the endothelial glycocalyx. The Src signaling mediated VE-cadherin phosphorylation leading to VE-cadherin internalization by activation of thrombin.

Rho GTPases are a class of small GTPases with a relative molecular mass of 20–30 kd and belong to the Ras superfamily. Currently 23 members of the Rho family have been identified, including RhoA, Rac and Cdc42 [87,88]. Rho/Rho-associated protein kinase (ROCK) and inflammatory response are accompanied by the whole process of acute lung injury. In LPS-induced acute lung injury, LPS is an integral part of the outer membrane of Gram-negative bacteria and is one of the key molecules involved in the initial stage of infectious inflammation [89]. Rho can be activated by a variety of cytokines and inflammatory mediators, and Rho/ROCK signaling can be activated by Thrombin, IL-1, TGF-β, endothelin-1 and angiotensin II (Figure 3). Previous studies have shown that pretreatment of ROCK inhibitor Y-27632 attenuates endotoxin-induced pulmonary edema and neutrophil migration [90,91]. It has been confirmed that ROCK can act on the VE-cadherin and catenin complex. The claudin-1 and Claudin-4 regulated the adhesion and tight junction of two cell connections. These cytokines and vasoactive substances are important substances that mediate a variety of inflammatory damage. The Rho/ROCK pathway is likely to integrate into the inflammatory signaling pathways mediated by these factors. Furthermore, there is a Rho/ROCK-mediated vascular endothelial injury pathway in ARDS.

### 5.2. Role of Claudins Signaling Pathway

Claudins, a transmembrane protein, has been found to have at least 27 members so far and is an important component of tight junctions (TJs). In tight junctions of epithelial cells, claudins are thought to be key integrin regulators for maintaining potential energy, ion transport selectivity, and the epithelial-endothelial transport mechanisms [92,93]. The complex and tightly linked structures directly affect the permeability of transport ion selective channels and are essential for maintaining barrier function. Disorders in Claudins disrupt barrier function, enhance transport permeability, and further aggravate pulmonary edema and inflammatory responses [94]. When lung tissue is subjected to ischemia/anoxia stimulation, the absence of oxygen during oxidative phosphorylation results in depletion of mitochondrial ATP, and a decrease in cellular energy can disrupt N^+^-K^+^-ATPase that maintains cytoplasmic ion balance, leading to cellular edema [95]. At this time, the pathogenesis of lung injury may be: on the one hand, it can affect the active transport of alveolar fluid by inhibiting the lung water scavenging ability of sodium channels, N^+^-K^+^-adenosine triphosphatase (NKA) and aquaporin (AQP) existing on AEC I/II. However, Claudins, which affect the connection between alveolar epithelium, especially the most directly related to the fluid flow between cells, aggravate pulmonary edema by changing the structure and function of the tight junctions between adjacent epithelial cells [96]. Otherwise, the normal function of ion channels is maintained as the basis of the stable expression of Claudins in the pulmonary epithelial barrier. Studies have shown that the function of NKA and epithelial cell polarity related, and this polarity mainly depends on tight junction formation and E-cadherin of exogenous expression. NKA regulates RhoA-mediated actin polymerization, which activator of actin polymerization provides the necessary impetus for the displacement of the tight junction between the apical and baso-epicardial regions. In conclusion, NKA is crucial in the formation and maintenance of tight junctions, and its mechanism relies in part on intracellular ionic homeostasis and eventually epithelial integrity [97].

## 6. Therapeutic and Molecular Interventions

Preventing tissue hypoperfusion and maintaining adequate gas exchange are important in ARDS [98,99]. The specific management of ARDS includes improving gas exchange and correcting the underlying pulmonary injury. Many pharmacologic therapies, such as glucocorticoids, surfactants, antioxidants, and anti-inflammatory agents, have been evaluated in Phase II and Phase III trials for ARDS [100,101,102]. Although no statistically effective treatments for ARDS have been reported, some treatments may have induced responses in subgroups of patients. Supportive treatments for ARDS, including sedation, nutrition support, and prophylaxis against other infectious diseases, decreased the ARDS mortality rate in studies in the 1990s [26,103,104]. Lung-protective ventilation has been reported to prevent barotrauma and late pulmonary injury in in vitro studies. Many animal studies reported high tidal volumes with high inspiratory pressures, leading to histological changes with formation of hyaline membranes. In that study, severe local inflammatory responses were noted, which caused progression to respiratory failure. Lung-protective ventilation decreased ARDS mortality by 3% [105]. In early studies, patients who received mechanical ventilation with appropriate positive end-expiratory pressure (PEEP) experienced improved oxygenation and reduced hypoperfusion due to the opening of collapsed alveoli and decreased intrapulmonary shunting. Low PEEP may also decrease ventilator-associated pneumonia and prevent pulmonary injury [106]. In ARDS patient, PEEP was applied at ≥5 cmH_2_O [107,108]. The major benefit in this population is the improvement in gas exchange by preventing alveolar collapse. High PEEP levels decrease repetitive alveolar opening and closing, but could potentially promote pulmonary injury [108]. Current studies do not recommend the application of high PEEP as a routine initial treatment in the ARDS population [8,109].

### 6.1. Corticosteroid

The effects of anti-inflammatory treatments in ARDS are inconclusive despite several clinical and basic studies over many decades. In current studies, no pharmacologic therapy for ARDS has been reported to statistically improve the rates of morbidity and mortality. Several anti-inflammatory treatments, including inhaled corticosteroids, angiotensin-converting enzyme inhibitors, and peroxisome proliferator-activated receptor agonists, have been tested to control mortality in ARDS. Corticosteroids have been reported to improve lung compliance in in vitro studies [101,110,111,112]. The corticosteroids inhibited general inflammation by decreasing the production of prostaglandin via activation of annexin I, which is an anti-inflammatory protein that inhibits cytosolic phospholipase A2a to block the production of arachidonic acid [113,114]. Corticosteroids also induce MAPK phosphatase 1 to inhibit cytosolic phospholipase A2a via blocking MAPKs and MAPK-interacting kinase. In addition, corticosteroids inhibit c-Jun, Fos, and NF-κB signaling to reduce the release of cytokines to control systemic inflammatory responses. Due to the complex anti-inflammatory effects of steroids, they have been used to treat allergic responses, autoimmune diseases, and inflammatory diseases in clinical practice. In ARDS, the corticosteroids controlled pulmonary inflammation and improved oxygenation [115,116]. However, in some studies, the beneficial effects of the therapeutic corticosteroids were not statistically significant [117,118]. In the future, large sample, randomized, controlled trials are necessary to examine the effects and efficiency of corticosteroids.

### 6.2. Angiotensin

Angiotensin, a peptide hormone, is part of the rennin–angiotensin system that regulates vasoconstriction to maintain blood pressure via several mechanisms, such as sympathetic nervous stimulation and release of aldosterone [119]. Initially, angiotensinogen is produced and released into the plasma from the liver. It is cleaved by renin, which is produced by the kidneys, to form angiotensin I. Angiotensin-converting enzyme, which is distributed on the pulmonary capillary endothelium, cleaves angiotensin I to produce the octapeptide angiotensin II, which is equipped with several biological effects to mediate vasoconstriction via the specific angiotensin II receptor type 1 and type 2 [120,121]. Angiotensin-converting enzyme plays a critical role in regulating vasoconstriction and vessel permeability. In ARDS, pulmonary endothelial injury decreases the body’s ability to convert angiotensin I to II [26,122,123], leading to the theory of angiotensin II insufficiency in ARDS patients. Initially, inflammatory events induce acute lung injury and endothelial injury. Angiotensin-converting enzyme dysfunction leads to angiotensin II insufficiency and catecholamine resistance [26,123]. Angiotensin II insufficiency decreases NF-κB gene expression, which block the renin–angiotensin axis. In an animal study, treatment with recombinant angiotensin-converting enzyme 2 in wild-type and knockout mice partially prevented lung injury, and ameliorated acute lung injury in wild-type mice [124].

### 6.3. mTOR

The PI3K signal transduction pathway plays an important role in many pathophysiological processes such as cell proliferation, apoptosis, migration, vesicle transport and malignant transformation of cells. The PI3K/Akt/PKB signaling pathways and the regulation of apoptosis play an important role. Previous studies found that lipopolysaccharide induced rat alveolar type II epithelial cells can increase PI3K/Akt signaling pathway in Nedd4-2 protein and phosphorylated Akt and cAMP/cCMP related proteins regulate lung epithelial cell apoptosis performance, but significant increase the area of pulmonary edema and lung tissue damage [2]. At the same time, the study confirmed that Th17 cells were significantly decreased either through inhibition of mTORC1 or its upstream (PI3K/AKT) [125]. The mTOR pathway is activated by a variety of different classes of stimuli: it senses cellular energy levels by monitoring the cellular ATP:AMP ratio, insulin and signals from the Wnt pathway [126]. Following receptor engagement, PI3K as a messenger to activate downstream targets including the kinase Akt. Ras homolog enriched in brain (RHEB) plays critical roles in the activation of mTOR, a serine/threonine kinase that is involved in the activation of protein synthesis and growth. RHEB is controlled by the GTPase-activating protein (GAP) activity of a complex consisting of tuberous sclerosis complex 1 (TSC1) and TSC2. TSC2 is phosphorylated and inactivated by Akt and the TSC1–TSC2 complex negatively regulates the kinase mTOR. When this complex is phosphorylated by Akt or Erk1/2, its GAP activity is inhibited and RHEB is active, leading to the activation of mTORC1 [127]. These results indicate that focusing on mTOR to regulate immune cell-mediated inflammation could be a useful treatment target.

### 6.4. Neuromuscular Blockade

The neuromuscular blocking agents (NMBA) in sedation ARDS patients significantly improved the severe hypoxemia by decreasing patient-ventilator dyssynchrony, oxygen consumption and endogenous effort of breathing. The chest wall compliance was also improved under administration of NMBA. In a multicenter, double-blind trial [128], 340 ARDS patients was included and induced paralysis by either cisatracurium besylate. After received NMBA for 48 h, the hazard ratio for death at 90 days was reduced. The incidence of complications was not different between NMBA and placebo group. In addition, NMBAs have anti-inflammatory effects to mild control the inflammatory response. However, NMBA-induced paralysis may increase the risk of acquired neuromuscular weakness leading to difficultly weaning from mechanical ventilation and increase mortality [17,129,130].

### 6.5. Vasodilators

In some ARDS patients, the diffuse pulmonary vasoconstriction was found and contributed to change vascular permeability and induce severe hypoxemia. Administration of selective vasodilatations, such as inhaled nitric oxide (iNO), seem to improve gas exchange in ARDS patients. Inhaled nitric oxide, inducing pulmonary vasodilation and redistribution of pulmonary blood flow. The inhaled form minimized the adverse systemic hemodynamic effects. Many randomized controlled trials revealed transient improvement in oxygenation [102,131,132,133]. The similar result was reported in one meta-analysis [134]. The iNO may be a short-term therapy in severe ARDS patients, especially presenting hypoxemia and respiratory failure. Another inhaled pulmonary vasodilator agent, prostacycline, also improves oxygenation. However, the reduction of mortality in ARDS patients is no significant [135]. Although several pharmacologic therapies have been reported, they all had controversial effects or mediated only mild improvements to ARDS in clinical studies. In the future, randomized controlled trials are necessary to examine the safety and efficacy of novel pharmacologic agents.

## 7. Conclusions

In this review, we present the current clinical and basic research associated with the molecular regulation of inflammatory cells and cytokines in the pathogenesis of ARDS. Several concepts are worth reiterating. First, pneumonia and sepsis are the most common causes for ARDS. The Berlin criteria have improved our ability to predict ARDS-related mortality. Second, ARDS is characterized by 3 major phases. In the exudative phase, APCs trigger immune responses leading to epithelial and endothelial cell damage. Alveolar macrophages are important pulmonary APCs that interact with neutrophils via Toll-like receptors. Neutrophils cause NETosis and the release of growth factors and cytokines to induce vascular hyperpermeability, which causes alveolar edema. In the proliferative phase, the release of VEGF increases vascular permeability and exudation of protein-rich fluid. The ROS released downstream of inflammatory cytokines induce apoptosis and necroptosis in ARDS. In the fibroproliferative phase, some inflammatory responses are not resolved and develop into chronic inflammation, leading to fibrosing alveolitis with cystic changes by infiltrating macrophages, fibrocytes, fibroblasts, and myofibroblasts. The latest phase is characterized by remodeling of the lung architecture with fibrosis and honeycomb formation, which impair gas exchange. Finally, preventing tissue hypoperfusion and maintaining adequate gas exchange are critical in ARDS management. Although no effective treatments for ARDS have been reported, many pharmacologic therapies in Phase II and Phase III trials for ARDS are being continuously evaluated. The complicated mechanisms of molecular regulation in ARDS remain unclear. This review provides an overview of the recent clinical and basic molecular studies in ARDS, thereby constituting a strong foundation for the development of further therapeutic interventions.

## Figures and Tables

**Table 1:** The diagnostic criteria of the American-European Consensus Conference (AECC) and Berlin definitions.

	AECC Definition from 1994 [20]	Berlin Definition from 2012 [21]
Timing	Acute onset	Within 1 week of a known clinical insult or new/worsening respiratory symptoms
Chest imaging	Bilateral infiltrates seen on frontal chest radiograph	Chest X-ray or CT scan: Bilateral opacities not fully explained by effusions, lobar/lung collapse, or nodules
Origin of edema	Pulmonary artery wedge pressure ≤18 mmHg when measured, or no clinical evidence of left atrial hypertension	Respiratory failure not fully explained by cardiac failure or fluid overload; objective assessment (e.g., echocardiography) required to exclude hydrostatic edema if no risk factor presents
Oxygenation	Acute lung injury criteria: PaO_2_/FiO_2_ ≤ 300 mmHg (regardless of PEEP level)	Mild ARDS: 200 < PaO_2_/FiO_2_ ≤ 300 with PEEP or CPAP ≥ 5 cmH_2_O
	ARDS criteria: PaO_2_/FiO_2_ ≤ 200 mmHg (regardless of PEEP level)	Moderate ARDS: 100 < PaO_2_/FiO_2_ ≤ 200 with PEEP ≥ 5 cmH_2_O
		Severe ARDS: PaO_2_/FiO_2_: ≤ 100 with PEEP ≥ 5 cmH_2_O

PEEP: positive end-expiratory pressure, CPAP: continuous positive airway pressure.

## Abbreviations

AECC	American-european consensus conference
AJ	Adherens junction
APC	Antigen-presenting cell
APCs	Antigen-presenting cells acute respiratory distress syndrome
AQP	Aquaporin
ARDS	Acute respiratory distress syndrome
CPAP	Continuous positive airway pressure
FiO_2_	Inspiratory oxygen fraction
IL	Interleukin
iNO	Inhaled nitric oxide
MLC	Myosin light chain
MLKL	Mixed lineage kinase domain-like protein
MLCK	Myosin light chain kinase
MLCP	Myosin light chain phosphatase
NET	Eutrophil extracellular trap
NF-κB	Nuclear factor-κb
NKA	N^+^-K^+^-adenosine triphosphatase
PaO_2_	Arterial oxygen tension
TNF	Tumor necrosis factor
PEEP	Positive end-expiratory pressure
RIP3	Receptor-interacting protein kinase 3
ROS	Reactive oxygen species
S1P	Sphingosine-1-phosphate
TGF	Transforming growth factor
TJs	Tight junctions
TNF	Tumor necrosis factor
VEGF	Vascular endothelial growth factor
